# Supplementary material for: Efficient Gaussian process-based motor hotspot hunting with concurrent optimization of TMS coil location and orientation
Source: PLoS Comput Biol. 2026 Feb 17;22(2):e1013994. doi: 10.1371/journal.pcbi.1013994 (PMC12928572; doi:10.1371/journal.pcbi.1013994)
Supplement: Attachment — Submitted filename: ResponseToReviewers.pdf [file pcbi.1013994.s001.pdf]

Reviewer #1

General Response:

We thank the reviewer for their thoughtful and constructive feedback. Their comments allowed us to clarify and refine our claims, ensuring the manuscript more accurately reflects the scope and implications of our work. The main revisions include:

- **Correction of overstated claims:** We revised the section on rTMS target selection to clarify that advances in motor hotspot identification improve dosing precision indirectly, by providing more reliable motor threshold estimates, rather than by directly solving the issue of non-motor targets.
- **Clarification on broader applicability:** We now clearly state that applications beyond the motor cortex require additional readouts, such as TMS-EEG, and have added references to support this point.
- **Discussion of clinical implementation:** We expanded on the practical challenges of translating the proposed framework into routine practice. This includes acknowledging the need for automation and CE-certification, while noting future plans for further software development. We also recognized the limitations for current researchers without such systems, positioning this as an important direction for future work.

These changes improve the manuscript's accuracy, balance, and clinical relevance. We are grateful to the reviewer for highlighting areas where our framing could be refined, which has led to a clearer and more nuanced presentation of the contributions and limitations of our study.

Below we provide point-by-point responses to each of the comments, along with details of the corresponding changes made to the manuscript.

1. **Reviewer Comment:** „A systematic review found that over 90% of studies determine rTMS intensity based on motor cortex stimulation, even when the intended cortical target is elsewhere (e.g., the dorsolateral prefrontal cortex) [83]. Our method has the potential to improve rTMS target selection by enhancing spatial precision and reproducibility, ultimately leading to more effective neuromodulatory interventions.“ Here are two argments likely incorrect connected. A more efficient hotspot identification does not solve the problem of using it as a target elsewhere.

   Authors' Response: We thank the Reviewer for identifying this incorrect statement. We have corrected it accordingly.

   Change(s) in Manuscript:
   Page 12, Lines 290-296:
   “*Enhancing the spatial and angular precision of TMS-based hotspot hunting has wide-ranging implications for neuroscience and clinical practice. More than 90 % of rTMS studies determine stimulation intensity based on motor cortex stimulation, even when targeting other regions such as the dorsolateral prefrontal cortex [83]. By*

improving hotspot localization, we provide a more precise and reproducible basis for estimating motor hotspots and, indirectly, motor thresholds, thereby enabling more accurate dosing in both research and clinical applications. In this way, advances in motor hotspot hunting extend well beyond motor cortex mapping, supporting the reliability and effectiveness of neuromodulatory interventions more broadly."

2. **<u>Reviewer Comment:</u>** Accordingly here „Importantly, the proposed method is not restricted to the motor cortex; it is broadly applicable to other TMS applications requiring parameter optimization, facilitating hotspot identification beyond the motor cortex." It should be clearly stated that other outreads such as TMS-EEG are needed.

   <u>Authors' Response:</u> We have expanded our discussion to address this important aspect.

   <u>Change(s) in Manuscript:</u>
   Page 12, Lines 301-303:
   "*For instance, combining this approach with alternative readouts such as electroencephalography could facilitate hotspot identification not only within the motor cortex but also beyond it, extending its utility across a wide range of neuroscientific and clinical studies [101-103].*"

3. **<u>Reviewer Comment:</u>** „is the absence of a CE-certified commercial neuronavigation system capable of defining the next target during neuronavigation" agreed. However two amendments might be helpful here. 1. An open source availability of the present programm might be helpful (or not??) just by advising „move coil xx cm towards…. and angulate it by xx ° clock- or counterclockwise…. Would the time consumption by manually feeding this program be worth the effort?. 2. Are there any advices for todays researchers without the program simply using conventional hotspot hunting for the moment?

   <u>Author's Response:</u> We thank the reviewer for these helpful suggestions. Regarding point 1, we are actively developing this approach further and plan to share a closed-loop version of the program in the future, which will enable automated guidance during hotspot hunting. Regarding the second aspect, we currently cannot provide specific recommendations for researchers relying on conventional hotspot hunting, but we acknowledge this as an important area for future work.

   <u>Change(s) in Manuscript:</u>
   Page 12, Lines 306-307:
   ""While our approach holds promise for clinical applications, its integration into routine practice remains challenging, primarily because it demands full automatization and CE-certification."

Reviewer #2

The authors propose a Gaussian process Bayesian-optimization framework that jointly optimizes both TMS coil position and orientation, and compare five acquisition functions on eight subjects' EMG data and on simulated data. They conclude that Thompson sampling is most robust. The idea is clear, the implementation should be open source, and the work has methodological and practical value. However, there are still some problems in the analysis in this paper. I recommend major revisions to strengthen validation, clarify methods, and temper claims before acceptance.
Individual points:

General response:

We sincerely thank the reviewer for their careful reading of our manuscript and for providing constructive feedback that has significantly strengthened our work. We have carefully addressed all of the raised concerns through clarifications, additional analyses, figure improvements, and extended discussion. The main revisions and additions include:

- **Corrections and clarifications:** Minor typographical issues were fixed, and figure legends and captions were expanded for clarity (particularly for Figure 3). We also clarified important details such as the role of k-means initialization, the inclusion of the first 10 samples, and the conditions under which random sampling is outperformed by acquisition functions.
- **Expanded methodological details:** We substantially elaborated on the generative modeling pipeline and simulation framework, including kernel choices, warping, stochastic sampling, and validation rationale. We also described the acquisition function parameters and provided details on reproducibility, with code and configuration files available in the public repository.
- **Comparisons to prior work:** A new, detailed discussion was added to highlight how our approach extends beyond previous Bayesian neuronavigation methods by jointly optimizing coil location and orientation in a continuous space, incorporating warping for physiological realism, and comparing a broader set of acquisition functions. This section explicitly addresses both mechanistic and quantitative distinctions.
- **Additional analysis and discussion:** We clarified the clinical feasibility of the simulation and experimental design, demonstrating that accurate hotspot estimation is achievable in practice with approximately 50 stimuli. We also discussed the time/cost trade-offs and advantages of robotic coil navigation.

Taken together, these revisions improve the transparency, rigor, and clinical relevance of the manuscript. We believe the updated version clearly demonstrates the methodological innovation and practical contributions of our work while appropriately situating it within the existing literature. We are grateful for the reviewer's valuable comments, which have helped us significantly improve the clarity and impact of the paper.

Below we provide point-by-point responses to each of the comments, along with details of the corresponding changes made to the manuscript.

1. **Reviewer Comment:** There is a redundant) in line 96.

   Authors' Response: Thank you for catching this error.

   Change(s) in Manuscript: The typo has been corrected.

2. **Reviewer Comment:** The results of random sampling is presented in Figure 1. However, in the corresponding analysis of Figure 1 (in text), the random sampling is not mentioned or compared with the other methods.

   Authors' Response: We thank the reviewer for pointing this out. We only compared random sampling briefly to UCB and EI but not in detail to all acquisition functions with all scenarios. Thus, a paragraph was added to fill in the missing information.

   Change(s) in Manuscript:
   Page 9, Lines 223-227:
   "*Random sampling exhibited an initial improvement when initialized with k-means points; however, this advantage diminished rapidly after approximately 10 additional evaluations. While initialization provided a brief performance gain, random sampling remained consistently outperformed by all acquisition functions once further samples were collected. Notably, in the absence of initialization, both EI and UCB underperformed relative to random sampling during early iterations, further emphasizing their reliance on a structured prior.*"

3. **Reviewer Comment:** The results in Figure 2 A do not present the data for the first 10 steps. The distribution of the starting point should have an influence on the performance of these methods and should not be omitted in the analysis.

   Authors' Response: Thank you for highlighting the omission of the first 10 steps in Figure 2A, and for your insightful comment regarding the influence of initialization on the methods' performance. We apologize for the lack of clarity on this point and have revised both the manuscript text and the figure presentation accordingly.
   The first 10 steps represent the initial points selected by the k-means algorithm, which are held constant across all acquisition functions to ensure a consistent starting baseline. Thus, the "starting point" for the adaptive acquisition strategies is the 11th sample. We recognize the importance of transparently presenting the full performance trajectory, including the effect of initialization.
   We have updated the manuscript to explicitly state that the initialization phase consists of 10 k-means-selected points, and clarified that this phase is included in the analysis for all acquisition functions. Below is an unclipped version of the figure that illustrates that the first ten samples are identical for all acquisition functions:

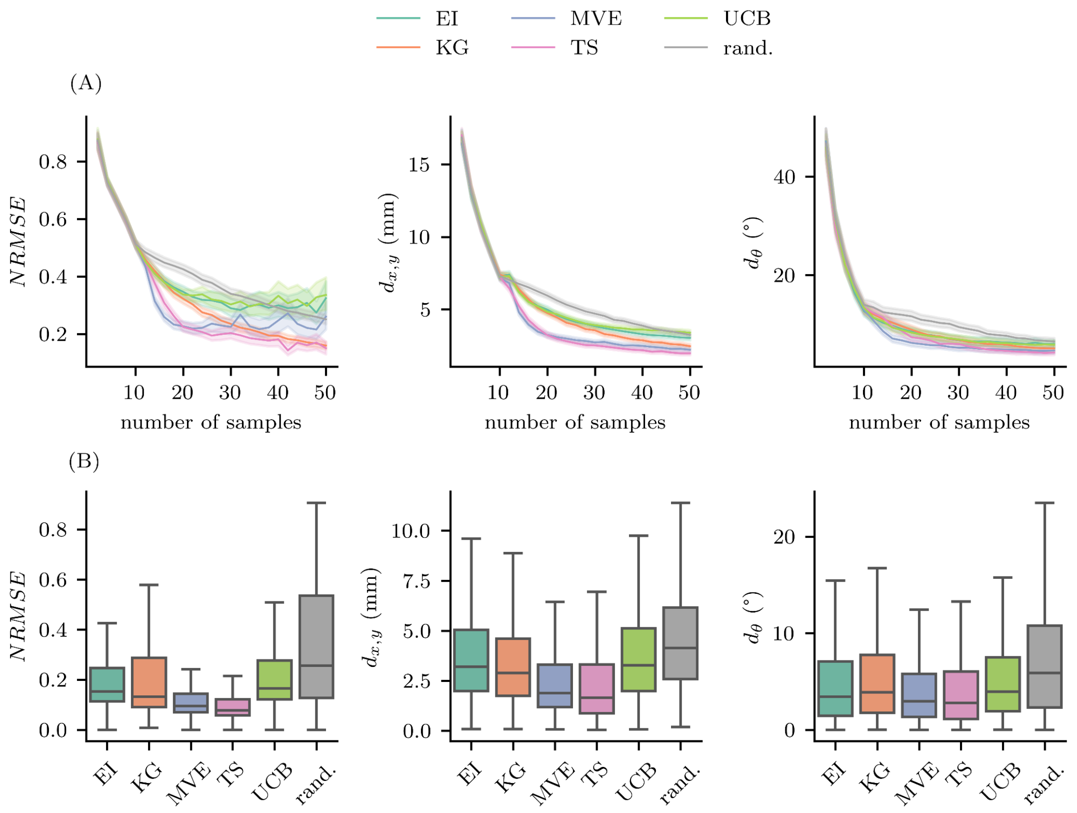

<u>Change(s) in Manuscript:</u>

Page 9, Lines 229-231:

"For all experiments, the first 10 data points were selected using k-means clustering and were identical for all acquisition functions. This standardized initialization provides a consistent basis for comparing subsequent adaptive sampling strategies."

4. **<u>Reviewer Comment:</u>** In the analysis of Figure 2B, you say
   "All acquisition functions outperform random sampling."
   But this is inconsistent with the performance of random sampling in Figure 1.

<u>Authors' Response:</u> Thank you for identifying the apparent inconsistency between the performance of random sampling in Figure 1 and our statement in the analysis of Figure 2B. This difference arises from the use of a k-means initialization strategy in the experiments underlying Figure 2B. In Figure 2B, all acquisition functions—including random sampling—start from an identical set of 10 k-means-initialized points. This initialization is designed to provide a representative coverage of the input space, ensuring a fair and consistent starting point for all methods.

In contrast, Figure 1 also compares conditions both with and without such an initialization. In the absence of structured initialization, random sampling can outperform guided methods.

To address this point and clarify for all readers, we have revised the manuscript text to explicitly state the role of k-means initialization in Figure 2 and that all acquisition functions outperform random sampling only under the given experimental setup.

Change(s) in Manuscript:
Page 10, Lines 243-244:
"Importantly, across the considered experimental setup with identical k-means initialization, all acquisition functions outperformed random sampling."

5. **Reviewer Comment:** The legend of Figure 3 is not obvious enough.

   Authors' Response: We thank the reviewer for this helpful comment regarding the clarity of the legend in Figure 3. To address this, we have revised the figure caption to explicitly define all visual elements. Additionally, we have clarified that the x and y axes correspond to a normalized 2D coil placement coordinate system projected onto the 3D scalp surface during stimulation. We further specify that both the ground truth and estimated hotspots are defined within this 2D coordinate space, extended into 3D when rotation is included. We believe these clarifications enhance the figure's readability and interpretation without requiring additional legend panels, thereby addressing the reviewer's concerns effectively.

   Change(s) in Manuscript:
   Page 10, Figure 3:
   "Each subplot illustrates an exemplary sampling pattern of a specific acquisition function after 30 stimuli within the circular spatial constraint on stimulus locations. The x and y axes represent a normalized 2D coil placement coordinate system, which is projected onto the 3D scalp surface at the location of stimulation. Small arrows mark stimulus locations, with their orientation indicating the coil rotation angle. Arrow color encodes the response amplitude, as shown by the adjacent colorbar labeled 'r' (response amplitude, arbitrary units). The red arrow denotes the ground truth hotspot, while the blue arrow indicates the estimated hotspot identified by the acquisition function. Both the ground truth and estimated hotspots are defined in this 2D coordinate space, extended into 3D when including coil rotation. All subplots share the same 10 initialization points. Acquisition functions illustrated are Expected Improvement (EI), Knowledge Gradient (KG), Max-Value Entropy Search (MVE), Thompson Sampling (TS), Upper Confidence Bound (UCB), and random sampling (rand.)."

6. **Reviewer Comment:** Please clearly states your advantage compared to the previous Bayesian neuronavigation papers. For example:
   a. https://doi.org/10.1016/j.neuroimage.2015.09.013
   b. https://doi.org/10.1016/j.neuroimage.2020.117082
   c. https://doi.org/10.1016/j.neuroimage.2017.04.001

   That should be a quantitative comparison that also looks into the mechanisms so that readers could really learn something and not just see yet another Bayesian method for the same without further information why and which one for what.

   Authors' Response: Thank you for this important and detailed comment. We appreciate the opportunity to clarify how our proposed method compares

quantitatively and mechanistically with prior Bayesian neuronavigation approaches for TMS hotspot hunting.

- The study by Meincke et al. (2016, DOI:10.1016/j.neuroimage.2016.06.051) introduced an automated closed-loop threshold estimation procedure using robotic coil positioning and testing approximately 100 discrete target locations. This approach required around 1000 stimulations, did not optimize coil orientation, and used a threshold-based algorithm without an explicit probabilistic surrogate model. While automating the hotspot search, the method is limited by its reliance on a fixed grid of stimulation sites and lack of joint spatial-angular parameter optimization, which results in less flexible target exploration and a higher demand for stimulations.
- Tervo et al. (2020, DOI:10.1016/j.neuroimage.2020.117082) applied Gaussian Process Regression as a surrogate probabilistic model within a Bayesian optimization framework. They utilized a knowledge gradient acquisition function to guide an adaptive search. However, coil location optimization was restricted to a constrained cross pattern, with coil orientation optimized separately only at the identified best location, rather than jointly. This strategy improved sample efficiency relative to exhaustive methods and allowed for uncertainty quantification, but the parameter space coverage remained limited, reducing potential resolution and mechanistic fidelity regarding coil orientation dependencies.
- Harquel et al. (2017, DOI:10.1016/j.neuroimage.2017.04.001) also applied a GP-based Bayesian model with entropy-based sampling strategies but restricted the stimulation to seven discrete points, without free target selection and without incorporating coil orientation parameters. Their acquisition function strategy, while information-theoretic, does not fully address the exploration-exploitation trade-off optimally, leading to potentially inefficient or suboptimal sampling.

In contrast, our method uses a warped Gaussian Process regression within a Bayesian optimization framework that jointly optimizes coil position and orientation in a continuous, unconstrained parameter space. The warping accommodates physiological constraints by ensuring positive motor evoked potential amplitudes. We evaluate multiple acquisition functions beyond traditional entropy or knowledge gradient methods, including Upper Confidence Bound (UCB), Expected Improvement (EI), Thompson Sampling (TS), Knowledge Gradient (KG), and Max-Value Entropy Search (MVE), to identify the optimal exploration-exploitation balance for this application. This joint continuous optimization captures the complex neurophysiological dependencies of motor evoked potentials on coil location and orientation and achieves empirical convergence substantially faster and with fewer stimulations than prior methods limited by discretized search spaces or restricted parameter optimization.
Mechanistically, by explicitly modeling both spatial and angular coil parameters simultaneously, our approach better reflects the true underlying biophysics of neuronal excitability modulation by TMS, which is inherently multidimensional and continuous. Quantitatively, this translates into more precise hotspot identification with fewer stimuli, addressing the practical concerns of participant comfort, experimental

time, and result reproducibility. In summary, our method advances Bayesian TMS neuronavigation beyond the current state-of-the-art by expanding parameter optimization dimensionality, enhancing model physiological realism through warping and continuous parameterization, and improving Bayesian optimization efficiency with a broad evaluation of acquisition strategies.

We note that all three papers were already cited in the original manuscript. Additionally, we have incorporated a detailed discussion of these distinctions in the manuscript to explicitly guide readers in understanding the mechanistic and practical advantages underpinning method selection and application.

Change(s) in Manuscript:
Page 3, Lines 78-80:
"Additionally, prior Bayesian neuronavigation methods have often constrained coil location to discrete grids or predefined patterns and relied on acquisition functions that may be suboptimal, limiting both sampling efficiency and mechanistic insight."
Page 3, Lines 82-85:
"Unlike previous methods [59, 69, 70], which restrict coil orientation, confine the search to discrete locations, or tune parameters separately, our approach jointly explores position and orientation in a continuous parameter space. It further leverages a broader range of acquisition functions beyond entropy-based methods, resulting in more efficient sampling and faster convergence."

7. **Reviewer Comment:** The abstract mentions "generative models" for data augmentation, but the manuscript lacks specifics on model type, parameters, or validation. Describe the simulation pipeline in detail and address potential biases. Moreover, if possible, can you describe the parameters of your 5 acquisition functions?

   Authors' Response: Thank you for this constructive comment. We agree that a detailed description of the generative model, simulation pipeline, validation steps, and acquisition function parameters is critical for transparency and reproducibility.
   1. Generative Model and Simulation Pipeline
   We have clarified in the methods (see " 2.1 Gaussian processes" and "2. 4 Dataset and MEP simulation" sections) that, for each participant, we fit a warped Gaussian process (GP) to the full set of 300 measured TMS coil-configuration/MEP amplitude pairs. The kernel function used is a squared exponential (RBF) kernel, with amplitude and length scale parameters fit via maximum likelihood at each iteration, and a square-root warping function to ensure non-negativity. Stochastic MEP responses are simulated by sampling from the posterior mean and variance of the fitted GP. We updated the relevant section to make this process clearer.
   2. Potential Biases
   Gaussian process regression has been rigorously applied in the field of TMS-MEP modeling and motor cortex mapping, showing strong capabilities in capturing spatial variability of motor evoked potentials while providing principled uncertainty quantification. Due to the practical constraints in acquiring new experimental data, we rely on this established methodology and prior validations reported in the literature to

justify using a warped Gaussian process as a generative model for simulating MEP responses. Such approaches have been demonstrated to approximate expert-guided mapping protocols accurately and have been adopted in numerous studies for probabilistic interpolation and optimization of stimulation targets. While direct prospective validation of our specific simulations was not possible, the strong foundational usage of GPs in this domain supports the soundness of our simulation pipeline.

3. Acquisition Function Parameters

All five acquisition functions are described analytically in the manuscript, with explicit mention of their key parameters. For UCB, the trade-off parameter β_n is set to decay sublinearly with acquisitions.

EI, TS, KG, and MVE are implemented according to standard BoTorch routines, with Monte Carlo sample sizes, optimization routines, and random seeds fully documented in the public codebase. The full source code and pipeline—including all settings for acquisition functions and simulation—are publicly available at the mentioned GithHub repository. This repository provides all configuration files, random seeds, MC sample sizes, and optimizer settings used, enabling full reproducibility.

We hope these clarifications and additions fully address the reviewer's concerns. Thank you again for helping us improve the clarity and transparency of the study.

<u>Change(s) in Manuscript:</u>

Pages 6-7, Lines 170-176:

*"Additionally, for every possible parameter settings **s**—even if not present in the original dataset—a simulated stochastic response is generated by sampling from the Gaussian process posterior predictive distribution defined by the mean $\mu_r(s)$ and variance $\sigma_r(s)$ of the ground truth model. This probabilistic generative model captures both the expected MEP amplitude and its uncertainty at arbitrary stimulation settings, allowing realistic simulation of response variability informed by the empirical data. GP regression has been rigorously applied in the context of TMS-MEP modeling and motor cortex mapping in several prior studies [70–72]. Due to practical constraints preventing new data collection, we rely on these established approaches and their demonstrated validity to underpin our simulation pipeline."*

8. **<u>Reviewer Comment:</u>** There are previous models for synthetic TMS motor responses with population statistics out there. A comparison would be great. A test with several of some such models would be even better as they might also increase the n, which could be a bit higher.

   <u>Authors' Response:</u> Thank you for your valuable suggestion. We acknowledge that several published synthetic TMS motor response models incorporate detailed population statistics, including complex input-output curves and noise modeling. Notably, our synthetic data model differs in several important ways: by keeping stimulator intensity fixed, we avoid the need for modeling a sigmoidal input-output curve with log-linear noise; instead, noise is modeled only around the ground truth MEP values. This noise is fitted directly from biologically collected data, ensuring it reflects true physiological variability.

In principle, our Gaussian process-based generative model can handle larger numbers of stimulations per subject (higher n), which could increase effective dataset size. However, a key goal of our work is to demonstrate convergence and reliable hotspot hunting even with relatively low n, which is common in practical motor mapping tasks. While population-based or circuit-level models provide alternative synthetic data perspectives, our approach prioritizes data-driven realism validated on empirical measurements and efficient use of limited samples.
We agree that a formal comparison with some of these existing generative models would be highly informative and represents a promising avenue for future work. A comprehensive comparative evaluation is beyond the scope of the present study; however, we encourage future research to incorporate such models, which may enhance sample size and improve generalizability.

Change(s) in Manuscript:
No changes were made in the manuscript.

9. **Reviewer Comment:** The simulations use 300 stimuli and evaluate ~2800 test points—this is maybe unrealistic in clinical settings. Is it possible to discuss performance under <100 stimuli and the time/cost trade off in conjunction with robotic navigation.

Authors' Response: We thank the Reviewer for raising this important point regarding the number of stimuli and test points used in our simulations. To clarify, the 300 stimuli (collected in a previous motor mapping experiment) serve to define the ground truth model, which is based on a realistic representation of the motor map and is entirely feasible within clinical settings. The approximately 2,800 test points are not additional measurements but rather synthetic samples generated from the fitted models to quantify the accuracy of the model fit via NRMSE. Therefore, these test points do not represent actual clinical sampling but serve as a high-resolution evaluation framework.
Importantly, our results demonstrate that a high-quality fit can be achieved with approximately 50 stimulation points, which aligns well with the practical limitations and common practices in clinical TMS mapping protocols. Thus, the stimulation count and model performance align with realistic clinical feasibility.
Regarding the time/cost trade-off, the use of robotic navigation facilitates precise and efficient coil positioning, potentially reducing setup time and increasing reliability. Incorporating adaptive sampling strategies, such as those tested here, further reduces the total number of stimuli required for accurate hotspot localization, making this approach both clinically practical and cost-effective.
We also added a paragraph in the discussion that clearly states that we meet clinical standards and need approximately 50 stimulations for hotspot estimation.

Change(s) in Manuscript:
Page 12, Lines 310-315:
"The method proposed in the present work could be integrated into already existing toolboxes such Brain Electrophysiological recording and STimulation (BEST), building on their already powerful capabilities and further enhancing them by

providing improved hotspot identification and parameter optimization [105]. Our results indicate that approximately 50 stimulation points are sufficient to achieve accurate hotspot localization, aligning well with practical clinical and robotic neuronavigation settings."
